# Utilization and expenses of outpatient services among tuberculosis patients in three Chinese counties: an observational comparison study

**DOI:** 10.1186/s40249-019-0590-0

**Published:** 2019-10-04

**Authors:** Xuan-Xuan Wang, Jia-Ying Chen, Hui Jiang, An-Na Zhu, Qian Long, John S. Ji

**Affiliations:** 1 School of Health Policy and Management, Nanjing Medical University, No. 101 Longmian Avenue, Nanjing, 211166 Jiangsu Province China; 2 Center for Health Policy Studies, Nanjing Medical University, No. 101 Longmian Avenue, Nanjing, 211166 Jiangsu Province China; 3 Creative Health Policy Research Group, Nanjing Medical University, No. 101 Longmian Avenue, Nanjing, 211166 Jiangsu Province China; 4 Zhenjiang Center for Disease Control and Prevention, No. 9 Huangshan South Road, Zhenjiang, 212004 Jiangsu Province China; 5 Environmental Research Center, Duke Kunshan University, No. 8 Duke Avenue, Kunshan, 215316 Jiangsu Province China; 6 Global Health Research Center, Duke Kunshan University, No. 8 Duke Avenue, Kunshan, 215316 Jiangsu Province China; 7 Nicholas School of the Environment, Duke University, Durham, NC 27708 USA

**Keywords:** Tuberculosis, Outpatient care expenses, Outpatient services, Case-based payment, Global budget payment

## Abstract

**Background:**

The China-Gates TB project Phase II implemented case-based payment reform in three Chinese counties in 2014, designed specifically for patients diagnosed with Tuberculosis (TB). This study aimed to examine the changes in utilization and expenses of outpatient services before and after the reform implementation, among TB patients in the three counties in China.

**Methods:**

We collected quantitative data using surveys in 2013 (baseline year) and 2015 (final year). We used outpatient hospital records to measure service utilization and medical expenses of TB patients. We conducted qualitative interviews with local health authorities, officers of health insurance agencies, and hospital managers (*n* = 18). We utilized three focus group discussions with hospital staff and TB doctors and nurses. The *χ*^2^ tests and Mann-Whitney U tests were used to analyse quantitative data, and the thematic analysis using a framework approach was applied to analyse qualitative data.

**Results:**

Dantu and Yangzhong counties enacted TB-specific case-based payment method in 2014. Jurong County maintained global budget payment but raised the reimbursement rate for TB care. Compared to the baseline, the percentage of TB patients in Dantu and Yangzhong with eight or above outpatient visits increased from 7.5 to 55.1% and from 22.1 to 53.1% in the final survey, respectively. Jurong experienced the opposite trend, decreasing from 63.0 to 9.8%. In the final survey, the total outpatient expenses per patient during a full treatment course in Dantu (RMB 2939.7) and Yangzhong (RMB 2520.6) were significantly higher than those in the baseline (RMB 690.4 and RMB 1001.5, respectively), while the total outpatient expenses in Jurong decreased significantly (RMB 1976.0 in the baseline and RMB 660.8 in the final survey). Health insurance agencies in Dantu and Yangzhong did not approve the original design with outpatient and inpatient expenses packaged together, revealed by qualitative interviews. Furthermore, staff at designated hospitals misunderstood that health insurance agencies would only reimburse actual expenses. Many TB doctors complained about their reduced salary, which might be due to decreased hospital revenue generated from TB care after the payment method reform.

**Conclusions:**

The intended effect on cost containment of case-based payment was not evident in Dantu and Yangzhong. In Jurong, where the global budget payment system maintained with the reimbursement rate enhanced, we found an effect on cost containment, but the quality of TB care might be compromised. The TB-specific case-based payment method could be redesigned to combine payment on outpatient and inpatient expenses and to set an appropriate payment standard for TB care during a full treatment course. Local health insurance agencies have to provide explicit explanations on the payment method. TB care providers should be provided with proper incentives. Monitoring and evluaiton on the quality of TB care should be undertaken at regular intervals.

**Electronic supplementary material:**

The online version of this article (10.1186/s40249-019-0590-0) contains supplementary material, which is available to authorized users.

## Multilingual abstracts

Please see Additional file 1 for translations of the abstract into the five official working languages of the United Nations.

## Background

China has a high tuberculosis (TB) burden. Nearly 9% of global TB cases are in China, with an estimated incidence of 63 per 100 000 people in 2017 [1]. As a global strategy for TB control and recommended by the World Health Organization (WHO), the “Directly Observed Treatment, Short-course” (DOTS) strategy was initially introduced to China in 1991 and expanded across the entire country in 2002 [2]. The DOTS strategy contains five elements: political commitment, case detection using sputum microscopy among persons seeking care for prolonged cough, standardized short-course chemotherapy under proper case-management conditions including directly observed treatment, regular drug supply, and a standardized recording and reporting system that allows assessment of individual patients as well as overall program performance [3]. Remarkable successes, including decreasing incidence, averted deaths, and decreased drug resistance, has been achieved worldwide after implementing the DOTS strategy [4–6].

TB services in the majority of China are provided at TB dispensaries from provincial to the county level, most of which is an affiliated department of the Center for Disease Control and Prevention (CDC) [7]. In the “CDC model”, TB dispensaries are responsible for TB case detection, diagnosis, treatment, and case management. General hospitals and other health facilities are required to report and refer suspected TB cases to TB dispensaries. Hospitals only treated complicated and severe cases. Research found that instead of TB dispensaries, TB patients tended to visit general hospitals and other health facilities such as village clinics for diagnosis and treatment and general hospitals were financially disincentivized to refer TB patients to TB dispensaries. CDC DOTS services did not treat a large proportion of TB patients [8, 9]. Since 2000 an “integrated model” of TB services has been implemented in several provinces. In the integrated model, a general hospital called the designated hospital is required to provide diagnosis, prescription, and follow-up management of TB patients. Other health facilities such as township health centres refer suspected TB patients to the designated hospital. TB dispensaries continue to provide public health care, including case reporting, defaults tracing, and health education [7, 10]. It is assumed that the integrated model could shorten health system delay. But based on modeling of TB control under different scenarios, researchers worried that the quality of care provided by the designated hospitals might not match or exceed that by the CDC DOTS services [11].

Currently, there are three basic health insurance schemes in China: Urban Employee Basic Medical Insurance (UEBMI), Urban Resident Basic Medical Insurance (URBMI), and New Rural Cooperative Medical Scheme (NCMS) [12]. China almost achieved universal insurance coverage, but the benefits remain shallow [13]. In general, these three basic health insurance schemes primarily cover inpatient services with a relatively low proportion of outpatient services reimbursed [14]. Researchers have found that registers under the UEBMI have the highest reimbursement rate for TB outpatient care, while URBMI and NCMS coverage did not provide adequate financial protection for the use of TB outpatient services [15]. After the initiation of China’s health care reform in 2009, medical expenditures were still on rapid escalation, which could be due to improper incentives for hospitals and doctors [16].

Along with China’s economic reform in the 1980s, hospitals were given autonomy and became underfunded [17, 18]. In response, hospital directors adopted bonus systems, rewarding doctors according to the volume of health services they provided and the revenues they generated [19]. Meanwhile, fee-for-service (FFS) was the predominant payment method in China, where the government retrospectively reimbursed hospitals for the specific item provided [20, 21]. Consequently, the profit motives for hospitals and doctors were aligned [22]. Doctors were incentivized to induce unnecessary health demand, prescribe expensive imported medications, and overuse high-technology diagnostic tests to generate more revenues [23]. Medical expenditures were, in consequence, growing rapidly [24]. Studies also found that TB designated hospitals had low adherence to the national guideline for TB diagnosis and treatment and intended to over-provide TB services, including over-prescription of second-line anti-TB drugs and extra tests [25].

To curb the excessive growth of medical expenditures, the Chinese Government has experimented several payment methods since the early 2000s, including a global budget, capitation, and case-based payment [18]. The global budget payment system is a fixed budget assigned to cover aggregate expenditures during a given period [26, 27]. In an empirical study conducted in Taiwan, the researcher found that the global budget payment system provided only blunt incentives to hospitals to control medical resource utilization [28]. In another study examining the payment methods in Taiwan, the results demonstrated that global budget payment system controlled the growth of medical expenditures, but inefficient service provision may occur, as no direct incentives for efficiency were employed and providers may prescribe drugs and services with positive profit margins [29]. The capitation refers to the scheme where providers are paid a fixed amount per enrolled person to provide a defined package of services during a given period [20]. In a payment reform of capitation implemented in Changde City of Hunan Province, researchers found that the capitation model reduced inpatient out-of-pocket cost but had an insignificant effect on the total inpatient cost [30]. Case-based payment is another alternative payment method to FFS, where payers gave providers a predetermined amount covering all health care services per case [22, 26]. International experience indicates that case-based payment can control medical expenditures, as hospitals can retain savings by reducing the use of improper drugs, tests, and examinations [31, 32]. However, pilot reforms identified adverse effects in China, patients received insufficient services, and those with complications were not enrolled in the scheme [22]. Although the payment method reform is one core area of China’s health care reform, the impacts that various payment methods may have on the cost and utilization of medical services in Chinese hospitals have not been examined with enough efforts yet.

In 2008, the Chinese Ministry of Health issued the *China TB Prevention and Control Plan: Guideline for Program Planning and Implementation* (“China TB guideline” for short), which recommends that TB treatment should be provided through outpatient care for 6–8 months, while only severely TB patients with complications and/or comorbidities should be hospitalized [33]. According to both international and domestic guidelines, TB cases should be diagnosed by sputum smear and X-ray and given first-line anti-TB drugs. Second-line anti-TB drugs, extra examinations, and hospitalization provided to uncomplicated TB cases are highly likely unnecessary [34]. However, in the integrated model, the admission rate and inpatient expenditures were high among uncomplicated TB cases and extra TB services including liver protection drugs, second-line anti-TB drugs, and CT scan were over-provided [25, 35].

In our study, we selected Zhenjiang, located in Jiangsu Province in eastern China, as one of the three sites in China to conduct the Phase II of a program entitled “China National Health and Family Planning Commission and the Gates Foundation TB Project (Phase II)” (“China-Gates TB project Phase II” for short) [36]. This project was a collaboration between the Government of China and the Melinda and Bill Gates Foundation and implemented by the Chinese Center for Disease Control and Prevention. As a primary component in the China-Gates TB project Phase II, Zhenjiang implemented a case-based payment method in 2014, which was used to reimburse TB designated hospitals for providing TB care to stimulate these hospitals to adhere to treatment regimens and contain cost [36].

Distinct from current case-based payment systems implemented in China, our TB project sites in Zhenjiang was designed specifically for patients diagnosed of TB. No study has been conducted to examine the effect of such payment method on regulating the use and controlling the cost of outpatient care for TB treatment. Under this circumstance, we hypothesized that after implementing case-based payment for TB care, the total outpatient expenses per patient during a full treatment course might decline and the average outpatient visits per patient may approach to the standard of 6–8 visits. To test these hypotheses, we investigated the trend in utilization and expenses of outpatient services among TB patients in the three counties of Zhenjiang before and after implementing the case-based payment method. This study was specific to the Chinese condition, but countries faced with similar problems may find it useful. As China bears a very high burden of TB and we found mixed results of case-based payment, other countries may read and consider the issues addressed by this study when implementing similar models in their health systems.

## Methods

### Study design and setting

Our study used an observational before and after comparison design. The three counties under Zhenjiang’s administrative jurisdiction, i.e., Dantu (DT), Yangzhong (YZ) and Jurong (JR), were selected according to their gross domestic product per capita in 2012, representing the high-, middle- and low-level economic development within Zhenjiang, Jiangsu Province. A baseline survey and final survey was conducted in April 2013 and July 2015, respectively.

### Reforming policy for TB care

#### Reforming policy in DT and YZ

According to the national TB treatment policy, the first-line anti-TB medications and three sputum smear tests are provided free of charge across the full treatment course for TB patients [25]. Additionally, one chest radiography is provided free of charge to TB patients in Zhenjiang.

Effective January 2014, DT and YZ enacted case-based payment, designed and implemented exclusively for TB care. For each TB case, the designated hospital is paid a fixed amount of RMB 2400 (80% of RMB 3000) for providing outpatient care relevant to TB treatment during a full treatment course (Table 1). CDC has designed a package of TB-related services covered by the fixed amount. The program included TB patients receiving health care at designated hospitals and covered by the basic health insurance schemes, and excluded patients without any basic health insurance and mobile patients covered by health insurance in other cities. Before the case-based payment reform implementation, 40% of outpatient care expenses could be reimbursed among TB patients with resident health insurance in DT, while none could be reimbursed in YZ.

**Table 1:** Reimbursement rates and payment methods of URBMI and NCMS for outpatient care of Tuberculosis patients in Zhenjiang before and after the payment method reform

	Baseline (2013)		Final (2015)	
	Reimbursement rate of outpatient expenses	Payment method	Reimbursement rate of outpatient expenses	Payment method
Dantu	40%	Global budget	- RMB 3000 per case - Reimbursement rate ≥ 80%	Case-based payment
Yangzhong	Uncovered	Global budget	- RMB 3000 per case - Reimbursement rate ≥ 80%	Case-based payment
Jurong	a. 30% at Jurong People’s Hospital b. 20% at other city-level hospitals c. 10% at hospitals outside of Jurong	Global budget	a. 80% at Jurong People’s Hospital b. 20% at other city-level hospitals c. 10% at hospitals outside of Jurong	Global budget

*URBMI* Urban Resident Basic Medical Insurance, *NCMS* New Rural Cooperative Medical Scheme

URBMI and NCMS have been integrated into resident health insurance in Zhenjiang

The WHO recommended TB treatment regimens consist of an intensive phase (the first 2 months after treatment initiation) and a continuous phase (4 months for new patients and 6 months for relapsed patients) [37]. Effective simultaneously with the case-based payment reform in DT and YZ, a 100-yuan (RMB) nutrition subsidy and another 30-yuan (RMB) transportation subsidy were provided to each TB patient of 65 years or above per month during the intensive treatment phase, which were financed by the National Major Public Health Services Program.

#### Reforming policy in JR

In contrast to DT and YZ, JR maintained the global budget payment system for outpatient services provided at all district-level hospitals, including the TB designated hospital. The annual budget for outpatient care at district-level hospitals is calculated by multiplying the actual reimbursed amount in the previous year with 1.15 (a 15% annual growth rate of outpatient expenditures set for district-level hospitals in JR). Ninety percent of the monthly budget is paid to hospitals per month, and the remaining 10% is paid according to the performance evaluation at the end of each year. Hospitals can have a maximum of 10% savings annually.

In response to the case-based payment reform advocated by the China-Gates TB project Phase II, JR raised the reimbursement rate of outpatient expenses for TB treatment from 30 to 80% at the TB designated hospital. Besides, a previous policy was withdrawn, which set a 120-yuan (RMB) payment standard of average expenses per outpatient visit in JR. The same policy on nutrition and transportation subsidies was implemented in JR starting from 2014.

### Data collection

#### Quantitative data

In both baseline and final surveys, patient survey was conducted in each county to investigate service utilization and medical expenses related to TB diagnosis and treatment. The probability proportional to size (PPS) method was adopted to select 90 patients per county, for a total of 270 TB patients to be sampled in the baseline and final survey, respectively. The inclusion criteria were that TB cases were uncomplicated, not drug-resistant, and had completed the full treatment course before April 2013 in the baseline and before July 2015 in the final survey. A total of 263 respondents participated in the baseline and 250 in the final surveys, respectively.

We collected hospital records of outpatient visits of TB patients from designated hospitals in both baseline and final surveys, including the number of outpatient visits, itemized expenses, and total expenses. In data collection, we provided name lists to designated hospitals, which contained information on the name, identity card number, sex, age, and the date of diagnosed as uncomplicated TB. Upon receiving this, hospital staff searched hospital outpatient information systems and generated hospital records of outpatient visits.

#### Qualitative data

In the final survey, 18 individual face-to-face interviews were conducted, including three managers of TB designated hospitals, five leaders from health bureaus, five from CDCs, and five from health insurance agencies at different levels who were mainly responsible for TB-related issues. These key informant interviews were used to explore the implementation of the project and the current challenges of the payment method reform for TB care. Besides, three focus group discussions were organized with medical staff from TB designated hospitals to obtain information on the payment method reform from the medical staff’s perspectives. There were around six interviewees in each focus group, including at least three doctors and one nurse from the department of infectious diseases, and staff from the hospital administrative office who were familiar with TB-related issues.

Documents on the health insurance policy and subsidies related to TB patients were collected from health bureaus and health insurance centres at different levels in both baseline and final surveys.

### Data analysis

#### Quantitative data

In both baseline and final surveys, some respondents had not completed the full treatment course before the survey commencement. We deleted from the datasets cases of respondents who were diagnosed as TB less than 6 months prior to our survey commencement. After deleting such cases, the total number of respondents included in the analysis was 259 in the baseline survey and 202 in the final survey. We linked hospital records of outpatient visits to patient survey datasets with patient name as the identifier. We successfully linked data of 230 and 174 respondents in the baseline and final survey, respectively.

Among the TB patients included in the study, 133 TB patients (57.8%) in the baseline survey and 111 (63.8%) in the final survey reported that they had been hospitalized for TB treatment. Because separate payment reform policies were implemented for TB outpatient and inpatient care fee, this study focused on the policy relevant to outpatient services and used the frequency of outpatient visits, average outpatient visits, total outpatient expenses and itemized expenses as key indicators. The *χ*^2^ tests were used to test differences in the frequency of outpatient visits between the baseline and final survey in each county. Mann-Whitney U tests were used to test differences in average outpatient visits, total outpatient expenses and itemized expenses between the baseline and final survey in each county. As once-a-month outpatient visit and the range of 6–8 visits during a full treatment course are recommended by the China TB guideline, the frequency of outpatient visits was used in this study to indicate the utilization of TB outpatient services. Because information collected from hospital records was after the date of diagnosed as TB for each included TB case, the frequency of outpatient visits could reflect whether designated hospitals adhered to the TB guideline during the full TB treatment course. It was grouped into < 6 visits, 6–8 visits, and > 8 visits. Analyses were performed in SPSS 22.0 (IBM Corp, Armonk, USA). A 5% significance level was used throughout the analyses.

#### Qualitative data

The approach used in the baseline survey to analysing qualitative data has been reported in other places [25]. The same thematic analysis using a framework approach employed in the baseline survey was performed in the final survey. All interviews were transcribed verbatim by one researcher and then the transcribed text was compared with the corresponding recording and notes taken during the interview by another researcher. Based on topic guides and transcripts, an analysis framework was developed to identify themes. All qualitative data were coded, sorted and categorized into the framework. NVivo 9.0 (QSR International, Melbourne, Australia) was used to manage qualitative data. As to policy analysis, two researchers worked closely in reviewing the policy documents and summarizing policy on payment methods, reimbursement rates and subsidies relevant to TB patients.

### Ethical considerations

The study design and implementation of the China-Gates TB project Phase II was approved by the Research Ethics Committee of the China CDC. Standard procedures were followed to obtain informed consent from each participant before their participation in the interview.

## Results

### General characteristics of TB patients

Among respondents in the baseline survey, no obvious differences were found in sex, age, or type of health insurance between the 230 respondents included in the linked dataset and the 29 excluded. Similarly, among respondents in the final survey, no obvious differences were found in sex, age or type of health insurance between the 174 respondents included in the linked dataset and the 28 excluded.

As presented in Table 2, over 70% respondents in the baseline and final survey were male patients. Most respondents had primary school education or below (55.2 and 64.9% in the baseline and final survey respectively). More than 80% respondents in both surveys had resident health insurance and around 90% respondents resided in rural area. Nearly 74% respondents in both surveys were new TB patients. The differences in age, employment and income were statistically significant between patients in the baseline and final survey. The mean age in the final survey was 62.6 years, compared to 59.2 years in the baseline survey. Fewer respondents in the final survey were employed or farmer. The average annual household income per capita in the final survey was lower than that in the baseline, with a difference of around RMB 2700.

**Table 2:** Demographic and socioeconomic status of Tuberculosis patients by survey period (percentage)

	Total		Dantu		Yangzhong		Jurong	
	Baseline	Final	Baseline	Final	Baseline	Final	Baseline	Final
	*n* = 230	*n* = 174	*n* = 80	*n* = 49	*n* = 77	*n* = 64	*n* = 73	*n* = 61
Sex								
Men	71.3	70.7	68.8	79.6	74.0	67.2	71.2	67.2
Women	28.7	29.3	31.3	20.4	26.0	32.8	28.8	32.8
Age								
Mean (years)	59.2	62.6*	57.8	60.5	60.0	66.0*	60.1	60.8
*Age group*								
< 30	6.1	5.2	3.8	8.2	7.8	3.1	6.8	4.9
30–59	36.1	28.7	45.0	30.6	31.2	23.4	31.5	32.8
≥ 60	57.8	66.1	51.3	61.2	61.0	73.4	61.6	62.3
Education level								
Primary school and below	55.2	64.9	47.5	49.0	50.6	75.0*	68.5	67.2
Middle school	34.8	24.1	42.5	36.7	33.8	21.9	27.4	16.4
High school	7.4	5.7	10.0	6.1	10.4	1.6	1.4	9.8
College and above	2.6	4.6	0.0	6.1	5.2	1.6	2.7	6.6
Missing	0.0	0.6	0.0	2.0	0.0	0.0	0.0	0.0
Occupational status								
Employed/farmer	46.1	39.1*	52.5	46.9	37.7	34.4*	47.9	37.7*
Unemployed	8.3	9.2	12.5	16.3	2.6	7.8	9.6	4.9
Retired	41.3	24.7	28.8	24.5	53.2	25.0	42.5	24.6
Disabled	3.9	12.6	6.3	10.2	5.2	15.6	0.0	11.5
Other	0.4	14.4	0.0	2.0	1.3	17.2	0.0	21.3
Income								
Mean (thousand yuan)	13.5	10.8*	13.8	12.5	16.2	10.0*	10.3	10.2
*Income group*								
Low	24.3	24.7	20.0	20.4	18.2	20.3	35.6	32.8
Middle	50.0	49.4	52.5	46.9	50.6	59.4	46.6	41.0
High	23.9	24.7	23.8	32.7	31.2	20.3	16.4	23.0
Missing	1.7	1.1	3.8	0.0	0.0	0.0	1.4	3.3
Health insurance								
None	2.2	5.2	0.0	4.1	6.5	3.1	0.0	8.2*
Resident health insurance	86.1	81.6	90.0	79.6	77.9	82.8	90.4	82.0
Other health insurance	11.7	13.2	10.0	16.3	15.6	14.1	9.6	9.8
Residence								
Rural	90.4	89.7	96.3	87.8	83.1	84.4	91.8	96.7
Urban	9.1	9.2	3.8	8.2	15.6	15.6	8.2	3.3
Unified	0.4	1.1	0.0	4.1	1.3	0.0	0.0	0.0
Diagnosis category								
New patient	73.9	73.6	81.3	69.4	67.5	62.5	72.6	88.5*
Relapsed patient	26.1	26.4	18.8	30.6	32.5	37.5	27.4	11.5

Data source: Patient surveys and hospital records

* *P* < 0.05

In DT, no differences in the general characteristics of TB patients between the baseline and final survey were statistically significance. But more relapsed patients were treated in the final survey (30.6%) than those in the baseline (18.8%). In YZ, compared with baseline, more disabled elderly patients with lower education level and much less household income per capita were treated in the final survey. In JR, there were more disabled patients without health insurance treated in the final survey. Besides, more new patients were treated in the final survey (88.5%) than those in the baseline (72.6%) in JR.

Most hospital managers reported in individual interviews that subsidies on nutrition expenditure and transportation fees contributed to improving TB patients’ adherence to treatment regimens and completion of the whole treatment course, especially among the poor and the elderly.

*“As far as I know, more than 150 TB patients including multi-drug resistant TB patients had received the nutrition and transportation subsidies. Nearly half of them were elderly patients. Rich patients did not care about the subsidies, but the poor were more willing to come to the hospital (than before). These subsidies contributed to completing the whole treatment course”* (a TB designated hospital manager, key informant interview).

### Outpatient service utilization

In Table 3, about 22% of TB patients in the final survey had 6–8 outpatient encounters, which was almost the same as that in the baseline. Different patterns were observed in the three counties. In DT, the percentage of TB patients who had fewer than six outpatient visits decreased from 86.3 to 30.6%, while the percentages of TB patients who had 6–8 outpatient visits and more than eight visits increased from 6.3 to 14.3% and from 7.5 to 55.1%, respectively. In YZ, nearly half of the patients had 6–8 outpatient visits in the baseline, but in the final survey only 31.3% patients had 6–8 outpatient visits. Patients having less than six outpatient visits almost halved (28.6% in the baseline and 15.6% in the final survey, respectively). The proportion of patients having eight outpatient visits rose from 22.1% in the baseline to 53.1% in the final survey. In JR, the proportion of patients who had fewer than six outpatient visits was 70.5% in the final survey, significantly larger than that in the baseline (24.7%). In the final survey only 9.8% patients had more than eight outpatient visits; however, in the baseline 63.0% had over eight visits. There was a slight increase in the percentage of patients having 6–8 outpatient encounters, rising from 12.3% in the baseline to 19.7% in the final survey.

**Table 3:** Outpatient service utilization per patient at designated hospitals during a treatment course by survey period and county (percentage)

	Total		Dantu		Yangzhong	Jurong		
	Baseline	Final	Baseline	Final	Baseline	Final	Baseline	Final
	*n* = 230	*n* = 174	*n* = 80	*n* = 49	*n* = 77	*n* = 64	*n* = 73	*n* = 61
Frequency of outpatient visits (visits)								
< 6	47.4	39.1	86.3	30.6*	28.6	15.6*	24.7	70.5*
6–8	22.6	22.4	6.3	14.3	49.4	31.3	12.3	19.7
> 8	30.0	38.5	7.5	55.1	22.1	53.1	63.0	9.8
Mean of outpatient visits (visits)	7.6	7.8*	3.2	9.1*	6.8	9.6*	13.2	4.8*

Data source: Patient surveys and hospital records

* *P* < 0.05

Substantial increase in the average outpatient visits was found in DT and YZ. The changes in DT and YZ were from 3.2 to 9.1, and from 6.8 to 9.6, respectively. But in JR the average outpatient visits had a sharp decline from 13.2 visits in the baseline to 4.8 visits in the final survey.

### Outpatient care expenses

As presented in Table 4, the total outpatient expenses per patient during the whole treatment course in the final survey was RMB 1986.6, significantly higher than that in the baseline (RMB 1202.6). Expenses on laboratory tests and other services in the final survey increased significantly, while expenses on image examinations decreased substantially. Expenses on medications also increased, but it was statistically insignificant.

**Table 4:** Outpatient care expenses per patient at designated hospitals during a treatment course by county and survey period (RMB)

	Total		Dantu		Yangzhong		Jurong	
	Baseline	Final	Baseline	Final	Baseline	Final	Baseline	Final
	*n* = 230	*n* = 174	*n* = 80	*n* = 49	*n* = 77	*n* = 64	*n* = 73	*n* = 61
Total expenditure	1202.6	1986.6*	690.4	2939.7*	1001.5	2520.6*	1976.0	660.8*
Lab tests	277.1	631.8*	228.9	964.6*	211.6	744.5*	399.0	246.2*
% of total expenditure	23.0%	31.8%	33.2%	32.8%	21.1%	29.5%	20.2%	37.3%
Image examinations	219.6	163.8*	246.7	30.2*	331.1	346.3	72.2	79.6*
% of total expenditure	18.3%	8.2%	35.7%	1.0%	33.1%	13.7%	3.7%	12.0%
Medications	467.1	648.5	99.8	1308.1*	342.7	450.0	1000.8	326.9*
% of total expenditure	38.8%	32.6%	14.5%	44.5%	34.2%	17.9%	50.6%	49.5%
Other services	238.9	542.6*	115.1	636.8*	116.1	979.9*	504.0	8.0*
% of total expenditure	19.9%	27.3%	16.7%	21.7%	11.6%	38.9%	25.5%	1.2%

Data source: Patient surveys and hospital records

* *P* < 0.05

In the baseline survey, expenses on medications accounted for the largest proportion of total outpatient care expenditure per patient during the whole treatment course (38.8%), followed in sequence by laboratory tests (23.0%), other services (19.9%) and image examinations (18.3%). Such expense structure changed in the final survey. Although expenses on medications still took the largest proportion of total expenditure (32.6%), expenses on laboratory tests followed closely (31.8%), and expenses on image examinations only took 8.2% of total outpatient care expenses.

Among the three counties in the final survey, the total outpatient expenses per patient were highest in DT (RMB 2939.7), which was nearly four times higher than that in the baseline. Increase in expenses on medications and laboratory tests in DT contributed to such leap of total outpatient expenditure. In YZ, the total outpatient expenses doubled in the final survey, where expenses on other services had the largest growth rate, followed by expenses on laboratory tests. JR was the only county where the total outpatient expenses upon completion of TB treatment experienced a significant downward trend (RMB 1976.0 and RMB 660.8 in the baseline and final survey, respectively). The decrease in expenses on other services and medications contributed the most to the decline of total outpatient expenses in JR.

One officer from the provincial-level health department expressed the view that health insurance agencies were unfamiliar with case-based payment and opposed to the original case-based payment design, in which a bundled payment for the total expenses during a whole TB treatment course was proposed instead of separate payment for outpatient and inpatient care expenses.

*“The case-based payment is a novel concept to health insurance agencies. They have been used to fee-for-service and did not approve the original payment design where outpatient and inpatient care expenses were packaged together. They only cared about actual expenses. For example, if the total expenses per patient are RMB 5000 and the actual expenses are lower than that amount, it seems that health insurance agencies only reimburse the actual expenses to hospitals”* (an officer from the provincial-level health department, key informant interview).

The majority of officer from health bureaus and CDCs expressed their worries that the case-based payment reform may not realize the intended effect in reducing TB patients’ disease burden, because the bonus system at TB designated hospitals did not change and doctors’ salary was still linked to the volume of health services they provided and revenues they earned.

*“The fee-for-service payment for TB care has been reformed, which is actually linked to medical staff’s salary. For example, when doctors from the TB department compare their salary with colleagues from the hepatitis department where fee-for-service has not been reformed, they (doctors from the TB department) will feel they have not earned as much as their colleagues”* (an officer of health bureau, key informant interview).

*“If the goal is to ease the burden of TB patients, government should figure out a way to provide sufficient subsidies (to TB designated hospitals). If salary or profit were not compensated to the level before the reform, doctors would certainly find out one way or another to make up for the loss (of profit)”* (an officer of CDC, key informant interview).

Many doctors from TB designated hospital complained of reduced salary, which might be due to the decreased hospital revenue generated from providing TB care after the case-based payment reform. TB doctors were not quite sure how health insurance agencies would pay to the hospital. One doctor expressed that health insurance agencies would only reimburse actual expenses, even though the policy stated that a fixed sum of RMB 2400 per case for outpatient services should be reimbursed by health insurance agencies to designated hospitals regardless of actual expenses. This might be a possible reason for providing more services reaching to ceiling of the agreed amount.

*“I know the policy (of the case-based payment), but according to our past experience they (health insurance institutions) will not reimburse 2400 yuan (per patient) to us (designate hospitals). If they do not implement the policy, we hospitals can do nothing about it. Given that we could not use up the 3000 yuan, CT was added into the service package in May this year”* (a TB doctor from a TB designated hospital, focus group discussion)*.*

## Discussion

This study discovered the unintended consequences of the reforming policy. Compared with baseline, the total outpatient expenses per patient during a full treatment course in the final survey increased significantly in DT and YZ, with the implementation of the case-based payment. However, in JR the global budget payment system maintained with the reimbursement rate enhanced for TB care. Compared with baseline, the total outpatient expenses per patient in the final survey declined significantly in JR. In DT and YZ, the intended effect on cost containment of the case-based payment method was not shown. But unexpectedly, the refined global budget payment system in JR exhibited positive effect on cost containment for TB care.

As recommended in the WHO framework, a full course of TB treatment should last for six to 8 months [38]. China TB guideline also recommends a once-a-month outpatient visit for TB patients. Accordingly, the average number of outpatient visits should fall into the range of 6–8 visits during a full treatment course among new and relapsed TB patients. As shown in our study, the average outpatient visits among TB patients in DT and YZ were less than four and seven visits in the baseline, respectively. The average outpatient visits in both counties increased to more than nine visits in the final survey, indicating over-provision of outpatient services. It is possible that the payment standard of outpatient expenses (RMB 3000) set in the case-based payment method was much higher than the actual expenses (RMB 1202.6 in the baseline). If the payment standard is set too high without effective measures, financial resources would be wasted [39]. As founded in the key informant interviews, the original plan in DT and YZ was to implement a bundled payment for the total expenses during a full TB treatment course instead of separate payment for outpatient and inpatient expenses. But health insurance agencies did not approve the original plan. The analysis on qualitative interviews indicated that health insurance agencies might only reimburse the actual expenses instead of paying the fixed sum of RMB 2400 for each TB case and the designated hospital might not be able to retain savings. In the qualitative interviews, many doctors from DT and YZ complained of their reduced salary due to the decreased hospital revenue generated from providing TB care after the implementation of case-based payment. Therefore, it is likely that doctors at DT and YZ were insufficiently motivated, driven to use up the fixed sum, and over-provided unnecessary TB outpatient services. In DT, the total outpatient expenses per patient in the final survey was quite close to the RMB 3000 cap. In YZ, the total outpatient expenses per patient doubled in the final survey. The designated hospitals treated more relapsed patients (having a longer treatment period and using more outpatient services) in both counties after the case-based payment reform. Based on the unintended consequences found in DT and YZ, systems thinking may improve the design of the TB-specific case-based payment [40]. Setting appropriate payment standard, providing proper incentives to TB care providers, and making concrete explanations on case-based payment to designated hospitals are critical to attaining the intended effect on cost containment of case-based payment.

According to China TB guideline, first-line anti-TB medications (free of charge) and X-ray examinations (less expensive than CT) should be provided to uncomplicated new TB cases [25], which may not incur heavy economic burden on TB patients. However, deviations from the national guideline were observed in DT and YZ after the case-based payment reform started. Expenses on medications increased by more than ten times in DT, and the expenses on laboratory tests accounted for the second largest proportion of total outpatient expenses in both DT and YZ. It is likely that second-line anti-TB medications, liver-protection drugs, and auxiliary laboratory tests might have been prescribed to a substantial proportion of TB patients in these two sites, which might load unnecessary burden on patients and their families [41].

Our study was different from previous ones in terms of the cost-containment effect of case-based payment. The outpatient expenses in DT and YZ increased substantially after the implementation of case-based payment, while other studies reported either reduction in expenses or no significant effect [42, 43]. One study using hospital records of urban insured employees showed that among patients with chronic obstructive pulmonary disease, case-based payment caused lower hospitalization costs and lower drug costs than fee-for-service [44]. It might be due to the reason that case-based payment is most commonly used in inpatient services and rarely applied to one single disease such as TB in this study [22]. For outpatient services, FFS mixed with capitation and pay-for-performance is more commonly used to reduce providers’ incentives for overprovision of health services [45].

Although the outpatient expenses for TB care in JR decreased significantly in the final survey, such change did not derive from implementing case-based payment. The global budget payment system maintained in JR. As a response to the case-based payment reform required in the TB project, JR raised the reimbursement rate of TB outpatient expenses from 30 to 80%. This can be interpreted as integration into the present health insurance system. As a result, the effect on cost containment of TB outpatient care was significant in JR, with the total outpatient expenses per patient decreasing from nearly RMB 2000 in the baseline to about RMB 660 in the final survey. However, such a reduction in outpatient expenses should be treated with caution. As the average outpatient visits decreased from 13.2 times in the baseline to only 4.8 times in the final survey, the quality of TB care at the designated hospital in JR might have been compromised. In addition, more patients uncovered by health insurance were treated in JR after the reimbursement rate was raised for TB cases. It is possible that pressure from health insurance fund caused such change, because the reimbursement rate rose by more than two times, while the annual budget for outpatient care did not increase with it. Further research should be conducted to understand whether such trend was attributed to the enhanced reimbursement rate of TB outpatient expenses.

The results of this study also indicated that more poor and elderly TB patients were included in the final survey than baseline, perhaps due to the provision of nutrition and transportation subsidies to TB patients. Hospital managers expressed in the individual interviews that subsidies on nutrition expenditure and transportation fees contributed to improving TB patients’ adherence to treatment regimens and completion of the whole treatment course, especially among the impoverished and the aged.

To the best of our knowledge, this study is the first to examine changes in utilization and expenses of outpatient services among Chinese TB patients after case-based payment was implemented, which was designed exclusively for TB care. A strength of our study is linking patient surveys with hospital records. To avoid recall bias, data on service utilization, and medical expenses all derived from hospital records. Another strength is to collect and analyse qualitative data, which could provide insight into how the payment methods were implemented from the perspectives of different stakeholders. Our study has several limitations. First, applying the before and after comparison study design, we cannot distinguish the effect of the payment method reform from other policy. Second, the results of this study might not be generalized to other places, as Zhenjiang is located in Jiangsu Province, which is a more prosperous region than the other parts of China. But Zhenjiang is one of the first-batch pilot cities in China undergoing a comprehensive health care reform, the findings of our study is informative to national policy rollout. Due to the limited sample, differences in socioeconomic variables between the baseline and final survey were not adjusted in analysing outpatient services utilization and expenditures, which is a limitation in interpreting the results. In future studies, larger samples should be collected to better inform the reforming policy effect on outpatient service utilization and expenses. This study was conducted only 1 year after the implementation of the case-based payment method, allowing us to see the immediate impact of policy change. For future efforts, it would be valuable to collect longitudinal data and examine the sustainability and long-term effects on cost containment and quality improvement of different payment methods in the three counties for TB care.

## Conclusions

In DT and YZ, the intended effect on cost containment of the TB-specific case-based payment method was not shown, and over-provision of TB outpatient services might happen. In JR, the refined global budget payment system unexpectedly exhibited a positive effect on cost containment for TB care. But the quality of TB care might be compromised in JR, where the average outpatient visits decreased significantly after the payment method reform. Based on findings in this study, the TB-specific case-based payment method could be redesigned to combine payment on outpatient and inpatient expenses together and to set an appropriate payment standard for TB care during a full treatment course. TB care providers should be provided with proper incentives. Local health insurance agencies have to provide explicit explanations on the payment method to designated hospitals, which may assure the latter that they can keep the savings by providing proper TB services. Monitoring and evaluation on the quality of TB care should be undertaken at regular intervals to ensure that quality of care is not compromised at designated hospitals.

## Additional file

Additional file 1: Multilingual abstracts in the five official working languages of the United Nations. (PDF 398 kb)

## Data Availability

The data generated during this study are not publicly available, due to the reason that sufficient information is contained to enable readers to identify sampled hospitals, but a de-identified analytical file is available from the corresponding author (Jia-Ying Chen, jychen@njmu.edu.cn) on reasonable request.

## Abbreviations

CDC: Center for Disease Control and Prevention
DOTS: Directly Observed Treatment, Short-course
DT: Dantu
FFS: Fee-for-service
JR: Jurong
NCMS: New Rural Cooperative Medical Scheme
PPS: Probability proportional to size
TB: Tuberculosis
UEBMI: Urban Employee Basic Medical Insurance
URBMI: Urban Resident Basic Medical Insurance
WHO: World Health Organization
YZ: Yangzhong
