# Supplementary material for: Utilization and expenses of outpatient services among tuberculosis patients in three Chinese counties: an observational comparison study
Source: Infect Dis Poverty. 2019 Oct 4;8:79. doi: 10.1186/s40249-019-0590-0 (PMC6777024; doi:10.1186/s40249-019-0590-0)
Supplement: Supplementary file 1 — Multilingual abstracts in the five official working languages of the United Nations. (PDF 398 kb) [file 40249_2019_590_MOESM1_ESM.pdf]

Translation of the abstract into the five official working languages of the United Nations

استخدامات و نفقات خدمات العيادات الخارجية لمرضى السل في ثلاث مقاطعات صينية: دراسة مقارنة رصدية

شوان شوان وانغ, ينغ تشين, هوي جيانغ, آنا زو, كيان لونغ, جون سي جي.

ملخص

خلفية نفذت المرحلة الثانية من مشروع بوابات الصين للسل اصلاح للأجور بناء على حالة في ثلاث مقاطعات صينية عام 2014, ونفذ خصيصا للمرضى المشخصين بالسل. سعت هذه الدراسة لمراقبة التغييرات في الاستخدامات والنفقات لخدمات العيادات الخارجية قبل وبعد تنفيذ الاصلاحات لمرضى السل في المقاطعات الثلاث في الصين.

الأساليب: قمنا بجمع بيانات كمية مستخدمين دراسات في 2013 (السنة البدائية) و2015 (السنة النهائية). استخدمنا سجلات مستشفيات العيادات الخارجية لقياس استغلال الخدمات والنفقات العلاجية لمرضى السل. واجرينا مقابلات نوعية مع سلطات الصحة المحلية, وموظفي وكالات التأمين الصحي, ومدراء المستشفيات (ن=18). اجرينا نقاشات مع ثلاث مجموعات صغيرة من موظفي المستشفيات و اطباء وممرضي السل. استخدمت اختبارات س2 و مان ويتني يو لتحليل البيانات الكمية واستخدم التحليل الموضوعي بنهج نظامي لتحليل البيانات الكمية.

نتائج: سنت مقاطعتا دانتو ويانغزونغ نظام دفع مخصص مبني على حالة لمرضى السل عام 2014. اما مقاطعة جورنغ فبقيت على نظام دفع شامل ورفعت معدل السداد لعلاج السل. وبالمقارنة مع البداية فان نسبة مرضى السل في مقاطعتي دانتو و يانغزونغ بمعدل زيارة 8 او اكثر للعيادات الخارجية قد ازدادت من 7.5% الى 55.1% ومن 22.1% الى53.1% في الدراسة الاخيرة على التوالي. اما مقاطعة جورنغ فشهدت العكس, فقد انخفضت من 63% الى 9.8%. وفي الدراسة الاخيرة وجد ان تكاليف العيادات الخارجية للمريض الواحد خلال فترة العلاج الكاملة في دانتو 2939.7 ين, و يانغزونغ 2520.6 ين كانت اعلى بشكل ملحوظ من التكاليف البدائية (690.4 ين, 1001.5 علي التوالي) بينما انخفضت تكاليف العيادات الخارجية في جورنغ بشكل ملحوظ(1976 في الدراسة الاولية و 660.8 في الدراسة الاخيرة). لم توافق وكالات التأمين الصحي في دانتو ويانغزونغ على التخطيط الاصلي لتكاليف العيادات الخارجية والداخلية معا المكشف عنها خلال المقابلات الكمية. لم توافق وكالات التأمين الصحي في دانتو ويانغزونغ على التخطيط الاصلي لتكاليف العيادات الخارجية والداخلية معا المكشف عنها خلال المقابلات الكمية. واشتكى العديد من اطباء علاج السل من تناقص رواتبهم والذي قد يكون نتيجة لتناقص دخل ايرادات المستشفى بسبب العناية بالسل بعد اعادة تشكيل نظام الدفع.

نتائج: لم يكن التأثير المقصود لاحتواء التكلفة المبني على حالة الدفع واضح في مقاطعتي دانتو و يانغزونغ. و في جورنغ حيث ان نظام الدفع للميزانية الشاملة بقي مع تعزيز معدل التعويض, وجدنا تأثيرا على احتواء التكلفة ولكن جودة علاج السل قد تتأثر. أمكن اعادة تخطيط نظام الدفع المبني عل حالة السل بالتحديد ليشمل الدفع تكاليف العيادات الداخلية والخارجية ووضع معيار دفع لعلاج السل خلال فترة العلاج الشامل. وعلى وكالات التأمين الصحي الشرح الوافي لنظام الدفع. ويجب مد معالجي السل بالحوافز اللازمة. ويجب ان يتم الاشراف والتقييم لجودة علاج السل في فترات منتظمة.

Translated from English version into Arabic by Lina Awad, Revised by Sarah Farhan, through

TRANSLATORS
WITHOUT BORDERS

中国三县肺结核患者门诊服务利用与费用情况：一项观察性前后对照研究

摘要

引言：中盖结核病项目二期于 2014 年在中国三县实施了专为肺结核患者设计的按病种付费改革。本研究旨在分析三县（丹徒、扬中、句容）肺结核患者在改革前后门诊服务利用和费用的差异。

方法：课题组分别于 2013 年（基线年）和 2015 年（终末年）开展现场调查，收集定量资料；利用门诊就诊记录分析肺结核患者的服务利用与医药费用情况；开展了针对当地卫生行政部门、医保机构负责人和院长的定性访谈（共计 18 人）；并组织了三场针对医院职工和结核科医护人员的焦点小组访谈。采用卡方检验和 Mann-Whitney U 检验分析定量数据，利用主题框架法分析定性资料。

结果：丹徒和扬中于 2014 年实施了肺结核按病种付费制。句容仍沿用原先的总额预付制，但提高了肺结核报销比例。终末调查中，丹徒和扬中接受过 8 次或更多门诊服务的肺结核患者占比明显高于基线，而句容则呈现出相反的变化。终末调查中，丹徒和扬中肺结核患者人均全疗程门诊总费用明显高于基线，但句容肺结核患者门诊总费用则较基线水平有明显下

降。定性访谈显示，丹徒和扬中医保机构并未采用最初的门诊住院费用打包设计。此外，定点医院职工误认为医保机构仍补偿实际费用。很多结核科医生抱怨收入下降，这可能是由于改革后结核科收入整体降低。
结论：在丹徒和扬中，按病种付费在费用控制方面的效果并未得到显现，并可能出现了过度提供肺结核门诊服务的现象，这可能是由不合理的支付标准、对支付方式的误解、对肺结核服务提供者不合理的激励方式所致。句容沿用了总额预付制，并提高了报销比。该举措对费用控制效果明显，但肺结核服务质量有可能受到影响。

Utilisation et dépenses des services ambulatoires chez les patients tuberculeux dans trois comtés chinois : étude comparative observationnelle

Xuan-Xuan Wang, Jia-Ying Chen, Hui Jiang, Anna Zhu, Qian Long, John S. Ji

Résumé
Contexte : La Phase II du projet China-Gates sur la tuberculose a mis en œuvre une réforme des paiements en fonction des cas dans trois comtés chinois en 2014, conçue spécifiquement pour les patients atteints de Tuberculose (TB). Cette étude visait à examiner les changements dans l'utilisation et les dépenses des services ambulatoires avant et après la mise en œuvre de la réforme chez les patients tuberculeux des trois comtés de Chine.
Méthodes : Nous avons collecté des données quantitatives à l'aide d'enquêtes menées en 2013 (année de référence) et en 2015 (dernière année). Nous avons utilisé les registres des consultations externes pour mesurer l'utilisation des services et les dépenses médicales des patients atteints de tuberculose. Nous avons mené des entretiens qualitatifs avec les autorités sanitaires locales, les responsables d'agences d'assurance maladie, et les directeurs d'hôpitaux ($n = 18$). Nous avons utilisé trois groupes de discussion avec le personnel de l'hôpital ainsi que les médecins et les infirmières spécialistes de la tuberculose. Les tests χ^2 et Mann-Whitney U ont été utilisés pour analyser les données quantitatives et l'analyse thématique utilisant une approche-cadre a été appliquée à l'analyse des données qualitatives.
Résultats: les comtés de Dantu et de Yangzhong ont adopté une méthode de paiement fondée sur des cas spécifiques à la tuberculose en 2014. Le comté de Jurong a maintenu le paiement du budget global mais a augmenté le taux de remboursement des soins antituberculeux. Par rapport aux valeurs initiales, le pourcentage de patients tuberculeux à Dantu et à Yangzhong ayant effectué huit visites ambulatoires ou plus a augmenté de 7,5% à 55,1% et de 22,1% à 53,1% dans la dernière enquête, respectivement. Jurong a connu la tendance inverse, passant de 63,0% à 9,8%. Lors de la dernière enquête, les dépenses totales en ambulatoire par patient au cours d'un traitement complet à Dantu (2939,7 RMB) et à Yangzhong (2520,6 RMB) étaient significativement plus élevées que celles de la base (690,4 RMB et 1001,5 RMB, respectivement), alors que les dépenses à Jurong ont considérablement diminué (1976.0 RMB dans la base de référence et 660.8 RMB dans la dernière enquête). Les agences d'assurance maladie de Dantu et de Yangzhong n'ont pas approuvé la conception initiale des dépenses pour les soins ambulatoires et hospitaliers conditionnées ensemble, révélées par des entretiens qualitatifs. En outre, le personnel des hôpitaux désignés a mal compris que les organismes d'assurance maladie ne rembourseraient que les frais réels. De nombreux médecins antituberculeux se sont plaints de la réduction de leur salaire, qui pourrait être due à la diminution des revenus hospitaliers générés par les soins antituberculeux après la réforme de la méthode de paiement.
Conclusions : L'effet recherché sur la maîtrise des coûts d'un paiement fondé au cas par cas n'a pas été évident à Dantu et Yangzhong. À Jurong, où le système de paiement du budget global a été maintenu avec un taux de remboursement amélioré, nous avons constaté un effet sur la maîtrise des coûts, mais la qualité des soins liés à la tuberculose pourrait être compromise. Le mode de paiement basé sur les cas spécifiques à la tuberculose pourrait être repensé pour combiner le paiement des dépenses ambulatoires et hospitalières et établir une norme de paiement appropriée pour les soins antituberculeux pendant un traitement complet. Les organismes locaux d'assurance maladie doivent fournir des explications explicites sur le mode de paiement. Les prestataires de soins antituberculeux devraient bénéficier de mesures incitatives appropriées. La surveillance et l'évaluation de la qualité des soins antituberculeux doivent être effectuées à intervalles réguliers.

Translated from English version into French by Sarah Merlin, Revised by Laurence Rapaille, through

Использование и расходы на амбулаторные услуги пациентов с туберкулезом в трех округах Китая: обсервационное сравнительное исследование

Сюань-Сюань Ван, Цзя-Ин Чэнь, Хуэй Цзян, Анна Чжу, Цянь Лун, Джон С. Цзи

Аннотация
Предпосылки: В рамках второго этапа осуществления проекта China-Gates по борьбе с ТБ была проведена реформа оплаты услуг в трех округах Китая в 2014 г., разработанная специально для пациентов с диагнозом "туберкулез" (ТБ). Цель данного исследования — рассмотреть изменения в использовании и расходах на амбулаторные услуги до и после принятия реформы у пациентов с ТБ в трех округах Китая.
Методы: Количественные данные были собраны с помощью исследований, проведенных в 2013 году (базисный год) и 2015 году (заключительный год). Для оценки использования услуг и расходов на медицинские услуги пациентов с ТБ использовали медицинскую документацию амбулаторных больных. Были проведены кволитативные интервью с местными органами здравоохранения, сотрудниками органов медицинского страхования и управляющими медицинскими центрами (n = 18). Состоялось три обсуждения в фокус-группах с сотрудниками медицинского центра, а также врачом и медсестрами, осуществляющими лечение туберкулеза. Для оценки количественных данных использовали тесты χ^2 и U-критерий Манна — Уитни, для оценки качественных данных применяли тематический анализ с использованием рамочного подхода.
Результаты: В 2014 году в округе Даньту и Янчжун был принят метод оплаты, специфичный и основанный на случаях ТБ. В округе Джуронг общий бюджет сохранился, но повысилась доля возмещения расходов на медицинские услуги. По сравнению с исходным уровнем процентное соотношение пациентов с ТБ в Данту и Янчжун с количеством амбулаторных приемов восемь и более увеличилось с 7,5 % до 55,1 % и с 22,1 % до 53,1 % в заключительном исследовании соответственно. В округе Джуронг сложилась совершенно иная тенденция, процентное соотношение снизилось с 63,0 % до 9,8 %. В заключительном исследовании общая сумма расходов на амбулаторные услуги на пациента в течение полного курса лечения в Даньту (2939,7 юань) и Янчжун (2520,6 юань) была значительно выше исходной суммы (690,4 юань и 1001,5 юань соответственно), в то время как общая сумма расходов на амбулаторные услуги в округе Джуронг значительно снизилась (1976,0 юань на исходном уровне и 660,8 юань в заключительном исследовании). Органами медицинского страхования в Даньту и Янчжун не был одобрен первоначальный проект пакета расходов на амбулаторные и стационарные услуги, как показали кволитативиыне интервью. Более того, персонал назначенных больниц не понял того, что органами медицинского страхования будут возмещены лишь фактические расходы. Многие врачи, занимавшиеся лечением пациентов с туберкулезом, пожаловались на снижение заработной платы, что могло произойти из-за снижения дохода для больницы от лечения больных туберкулезом после проведения реформы способа оплаты.
Выводы: Желаемый результат по ограничению расходов благодаря оплате в каждом конкретном случае в округах Даньту и Янчжун не был очевидным. В округе Джуронг, где всеобщая система оплаты осталась неизменной, а ставка возмещения увеличилась, эффект от ограничения расходов был обнаружен, но качество услуг по лечению пациентов с туберкулезом могло пострадать. Метод оплаты услуг по лечению больных туберкулезом в каждом конкретном случае можно пересмотреть, чтобы согласовать оплату расходов на амбулаторные и стационарные услуги и установить соответствующий стандарт оплаты услуг

по лечению больных туберкулезом в течение полного курса лечения. Местные органы медицинского страхования должны предоставить развернутые объяснения относительно метода оплаты. Поставщикам услуг по лечению больных туберкулезом необходимо предоставить соответствующие льготы. Следует регулярно осуществлять контроль и оценку качества медицинских услуг по лечению пациентов с ТБ.

Translated from English version into Russian by Veronika Demeshchyk, Revised by Alexander Somin, through

TRANSLATORS
WITHOUT BORDERS

Utilización y gastos de los servicios ambulatorios, entre pacientes con tuberculosis, en tres condados chinos: un estudio de comparación observacional

Xuan-Xuan Wang, Jia-Ying Chen, Hui Jiang, Anna Zhu, Qian Long, John S. Ji

Resumen
Antecedentes: En el 2014, el proyecto de TB China-Gates Fase II implementó la reforma de pagos que se basó en casos, en tres condados chinos, diseñada específicamente para pacientes diagnosticados con Tuberculosis (TB) El objetivo de este estudio consistió en examinar los cambios, en la utilización y los gastos de los servicios ambulatorios, antes y después de la implementación de la reforma, entre los pacientes con tuberculosis, en los tres condados de China.
Métodos: Obtuvimos datos cuantitativos de encuestas realizadas en el año 2013 (año de inicio) y 2015 (año de finalización). Utilizamos registros hospitalarios ambulatorios, para medir la utilización del servicio y los gastos médicos de los pacientes con tuberculosis. Realizamos entrevistas cualitativas, con autoridades sanitarias locales, funcionarios de agencias de seguros de salud y gerentes de hospitales (n=18). Creamos tres grupos de debate, con personal del hospital, médicos especializados en TB y enfermeras. Para analizar los datos cuantitativos, se utilizaron las pruebas de χ^2 y Mann-Whitney U para analizar datos cuantitativos, y el análisis temático mediante un enfoque de marco de trabajo se aplicó para analizar datos cualitativos
Resultados: Los condados de Dantu y Yangzhong establecieron el pago, basado en el costo específico de tratamiento por TB, en el año 2014. La ciudad de Jurong mantuvo el pago del presupuesto global, pero aumentó la tasa de reembolso por el tratamiento de TB. En comparación con el valor basal, el porcentaje de pacientes con tuberculosis, en Dantu y Yangzhong, con ocho o más visitas ambulatorias, aumentó del 7,5% al 55,1% y del 22,1% al 53,1%, en la encuesta final, respectivamente. Jurong experimentó la tendencia opuesta, disminuyendo del 63,0% al 9,8%. En la encuesta final, los gastos totales de ambulatorio, por paciente durante un curso completo de tratamiento en Dantu (RMB 2939.7) y Yangzhong (RMB 2520.6) fueron significativamente más altos que los de la línea de base (RMB 690.4 y RMB 1001.5, respectivamente), mientras que el total ambulatorio los gastos, en Jurong, disminuyeron significativamente (RMB 1976.0 en la línea de base y RMB 660.8 en la encuesta final). Las agencias de seguros de salud en Dantu y Yangzhong no aprobaron el diseño original, con los gastos de pacientes ambulatorios e internos a la vez, revelados por entrevistas cualitativas. Además, el personal de los hospitales designados malinterpretó que las agencias de seguro de salud sólo reembolsarían los gastos reales. Muchos médicos de TB se quejaron de un salario reducido, lo que podría deberse a la disminución de los ingresos hospitalarios, generados por la atención de la TB, después de la reforma del sistema de pago.
Conclusiones: El efecto deseado de control de gastos por la reforma del sistema de pago, basado en el costo específico del tratamiento, no fue notorio en las ciudades de Dantu y Yangzhong. En la ciudad de Jurong, donde se mantuvo el pago del presupuesto global, con un aumento en la tasa de reembolso, sí se evidencia un efecto en el control de gastos. Sin embargo, en este caso podría verse comprometida la calidad del tratamiento por TB. El método de pago basado en casos específicos de tratamiento por TB podría ser modificado, para unificar el pago de los gastos de pacientes

ambulatorios y hospitalizados, y también, para establecer una tarifa convencional apropiada para pacientes con TB, durante todo su tratamiento. Las agencias locales de seguros de salud tienen que proporcionar explicaciones explícitas sobre el sistema de pago. Los proveedores de atención médica relacionada a la TB, deberían contar con incentivos adecuados. Se debería supervisar y evaluar, con regularidad, la calidad del tratamiento de TB.

Translated from English version into Spanish by Silvina Emilce, Revised by María Luz Puerta, through

TRANSLATORS
WITHOUT BORDERS
